# Study of single and multidigit activation in monkey somatosensory cortex using voltage-sensitive dye imaging

**DOI:** 10.1117/1.NPh.4.3.031219

**Published:** 2017-05-27

**Authors:** Anna Wang Roe, Jeremy E. Winberry, Robert M. Friedman

**Affiliations:** a Zhejiang University, Qiushi Academy for Advanced Studies, Interdisciplinary Institute of Neuroscience and Technology, Hangzhou, China; b Oregon Health and Science University, Oregon National Primate Research Center, Division of Neuroscience, Beaverton, Oregon, United States; c Vanderbilt University, Department of Psychology, Nashville, Tennessee, United States; d The University of Chicago, Department of Organismal Biology and Anatomy, Chicago, Illinois, United States

**Keywords:** nonhuman primate, somatosensory cortex, optical imaging, voltage-sensitive dye imaging, motion, funneling

## Abstract

Toward the goal of understanding cutaneous sensory integration during manual behavior, we used voltage-sensitive dye (VSD) imaging to study the organization and dynamics of anesthetized monkey primary somatosensory cortex (SI) in response to single and multidigit tactile stimulation. We find that in both macaque and squirrel monkey SI, VSD reveals clear focal digit topography consistent with previous electrophysiological and intrinsic signal imaging studies. VSD also reveals interactions in SI in response to multidigit stimulation. With a tactile funneling paradigm in areas 3b and 1 in squirrel monkeys, VSD reveals two-digit induction of subthreshhold influences, consistent with lateral intracortical inhibition. In response to tactile apparent motion stimuli, VSD reveals preferential response to motion stimuli over static tactile stimuli in both areas 1 and 3b. Comparison of the response at different digit locations to “toward digit” stimuli suggests the presence of direction-selective response in area 1; however, further study is needed. These exciting results indicate that VSD constitutes a powerful tool for studying somatosensory cortical processing in nonhuman primates and should be further developed for future somatosensory studies in awake behaving monkeys.

## 1 Introduction

Voltage-sensitive dyes (VSDs) are ideal for revealing the spatiotemporal patterns of cortical population response at mesoscales (50 to 100-μm resolution) and high temporal resolution (millisecond). This has proven enormously useful for studying the dynamics of cortical activity in both anesthetized and awake monkeys.^1^^–^^4^ In the visual cortex, VSD has revealed spatiotemporal organization of retinotopic activation,^5^ stimulus-induced traveling waves,^6^ motion processing^7^ (in cat), binocular integration,^8^ contour integration and shape,^9^^,^^10^ and color encoding.^11^ VSD imaging has been highly instrumental for extending our understanding of visual feature representation and integration in monkey visual cortex. However, thus far, to our knowledge, VSD has not been used to examine somatosensory cortical processing in nonhuman primates.

A number of studies of primate somatosensory cortex have focused on the region representing the hand as hand use is a central and defining feature of primate behavior. Understanding the pattern and dynamics of cutaneous inputs from the hand during such manual behavior is lacking and is crucial for designing manual prosthetics.^12^^,^^13^ Previous work predicts that somatosensory cortical networks underlying different manual grasps are dynamic and reconfigure based on the type of grasp being performed.^14^^,^^15^ In our studies, based on evidence from resting state fMRI connectivity, electrophysiological spike correlation, and anatomical connectivity, we have demonstrated the presence of two axes of cutaneous digit integration in SI: an interareal connectivity that is digit specific and an intraareal connectivity that achieves cross-digit integration.^16^^,^^17^ We hypothesize that these two axes of connectivity underlie the tactile percepts associated with precision grip and power grasp, respectively. Such predictions could be explored with the development of VSD methods for studies in somatosensory cortex of awake behaving monkeys.

In this study, we present results from our initial examination of spatiotemporal response patterns in anesthetized macaque and squirrel monkey SI. Results are presented from studies on single digit and multidigit activations. Multidigit activations are studied using a two digit funneling paradigm and a multidigit apparent motion paradigm. Using VSD, we report new observations about spatial and temporal cortical integration of tactile information. We find that VSD is a robust method for studying primate somatosensory cortex, one which should be further developed for studies of tactile encoding and manual behavior in primates.

## 2 Materials and Methods

### 2.1 Surgical Procedures

All procedures were conducted in accordance with the National Institutes of Health guidelines and approved by the Vanderbilt University Animal Care and Use Committee and followed the guidelines of the National Institutes of Health Guide for the Care and Use of Laboratory Animals. Two macaque monkeys and three squirrel monkeys were used in these studies. Monkeys were anesthetized with ketamine hydrochloride (10  mg/kg)/atropine (0.05  mg/kg) and maintained with isoflurane anesthesia (0.8% to 2%) delivered in a 70∶30 $O_{2}/N_{2}O$ mixture. Animals were intubated and artificially ventilated. Lactated Ringer’s solution was infused intravenously (2 to 3  ml/kg/hr) to prevent dehydration during the course of the study. Arterial blood oxygen saturation and heart rate, electrocardiogram, end-tidal ${\mathrm{CO}}_{2}$, and respiration were monitored and maintained. Temperature (37.5°C to 38.5°C) was monitored and maintained via a circulating water blanket. Real-time monitoring was maintained from the time of induction of anesthesia until full recovery. A craniotomy and durotomy were performed while the animal was under a surgical plane of anesthesia to expose primary somatosensory cortex (SI).

### 2.2 Intrinsic Signal Optical Imaging and Electrophysiology

Detailed procedures for optical imaging of monkey somatosensory cortex have been previously published^18^^,^^19^ and will be described here briefly. Prior to optical imaging, a few exploratory electrode penetrations were made to confirm digit locations in SI. Cortex was stabilized using 4% agar and a coverglass, and signal optical imaging (OIS; 632-nm illumination) was performed to map digit regions of areas 3b and 1 in the squirrel monkey and areas 1 and 2 in the macaque monkey. Vibrotactile stimuli (8 Hz train of 20-ms indentations of 300  μm lasting for 3 s for intrinsic imaging) were presented to digit tips and were compared with a blank (no stimulus condition). In some runs, distal phalanges on multiple digits were stimulated in sequence or simultaneously. Stimuli were presented in blocks, where each block contained four to nine stimulus conditions presented in a randomly interleaved fashion. Imaging runs consisted of 20 to 50 trials of each stimulus condition. Trial averaging was used to improve signal to noise. Frame size was 256×256  pixels and represented up to 20  mm×20  mm of cortical area.

### 2.3 Voltage-Sensitive Dye Imaging

For VSD imaging, somatosensory cortex was stained with VSD RH-1691, 1692, or 1838 (Optical Imaging, New York). Dye was dissolved in artificial cerebrospinal fluid, and concentration of the dye was determined by visual inspection of solution color. Application of dye to cortex for up to 2 h resulted in successful staining in roughly 50% of these attempts. We found that RH-1692 was reasonably effective. In the squirrel monkey cortex, dye was placed on the cortex and replaced once or twice over the course of staining. In the macaque monkey cortex, application was applied by infusion with a minipump through a tube attached to a piece of artificial dura that covered the cortex during infusion. The craniotomy was covered during infusion to prevent the possibility of bleaching due to stray light. Following successful staining of cortex, a CCD camera (NeuroCCD 256, SciMeasure Analytical Systems, Inc., Decatur, Georgia) was positioned over the craniotomy. Images were collected with a Redshirt Imaging System running CortiPlex software (Redshirt Imaging, Decatur, Georgia). After staining, cortex was rinsed and covered with 4% agar and a glass coverslip. For VSD imaging, cortical exposure to 632-nm illumination was gated by an externally controlled shutter (Uniblitz, Vincent Associates, Rochester, New York) to minimize light exposure and dye bleaching. To image VSD fluorescence, the camera lens was outfitted with a 650-nm high-pass filter. The CCD camera acquired frames at 100 Hz with a 256×256-pixel array.

The imaging sequence was as follows [see Fig. 5(a)]: (1) each trial began with the illumination of cortex with 632-nm light (shutter open), (2) the CCD camera started acquiring frames 40 ms prior to digit stimulation, (3) digit stimulation conditions (see below) lasted from 20 to 360 ms (see below), (4) at 540 ms, the camera stopped collecting frames and the shutter was closed, and (5) an 8-s interstimulus interval separated trials.

Mapping of digit representation in SI was conducted by either vibrotactile stimulation (8 Hz train of 20-ms indentations lasting for 3 s for intrinsic imaging) or single tactile indentation of single digits (for VSD). We used two paradigms to explore multidigit interactions. In tactile funneling experiments (Fig. 4), there were four conditions: D1, D2, simultaneous D1+D2, and blank (no stimulation). In tactile motion experiments (Fig. 5), apparent motion stimuli across digit tips were presented; these consisted of sequential stimulation of multiple digits each with a 20-ms duration tap (e.g., D1-to-D2-to-D3) with stimulus onsets of 50, 70, and 90 ms, respectively. These were compared with other control conditions: (1) slow sequential tapping of D1, D2, and D3 at 50, 220, and 390 ms that do not induce motion percept, (2) simultaneous stimulation of multiple digits (D1, D2, and D3), (3) an indentation of a single digit, and (4) no stimulation. Each imaging run consisted of 45 to 80 trials of each stimulus condition presented in a random order.

### 2.4 Image Processing

To generate maps for visualizing the spread of activity and for making threshold measurements of the area of activation, the raw ΔF/F images were spatially filtered using a spatial Gaussian filter with a sigma of 8 pixels for 100 Hz images (256×256  pixels).^20^ With a functional map of the representation of the finger pads, region of interests (ROIs) were selected with respect to individual digits. Image analysis was performed using custom MATLAB code (MathWorks Inc., Natick, Massachusetts). ROIs were centered on digit activation sites using superpixels 10×10  pixels in size and timecourses plotted.

## 3 Results

### 3.1 Can Voltage Sensitive Dyes Reveal Somatosensory Topographic Maps in Monkey SI?

#### 3.1.1 Macaque monkey

**Fig. 1:**
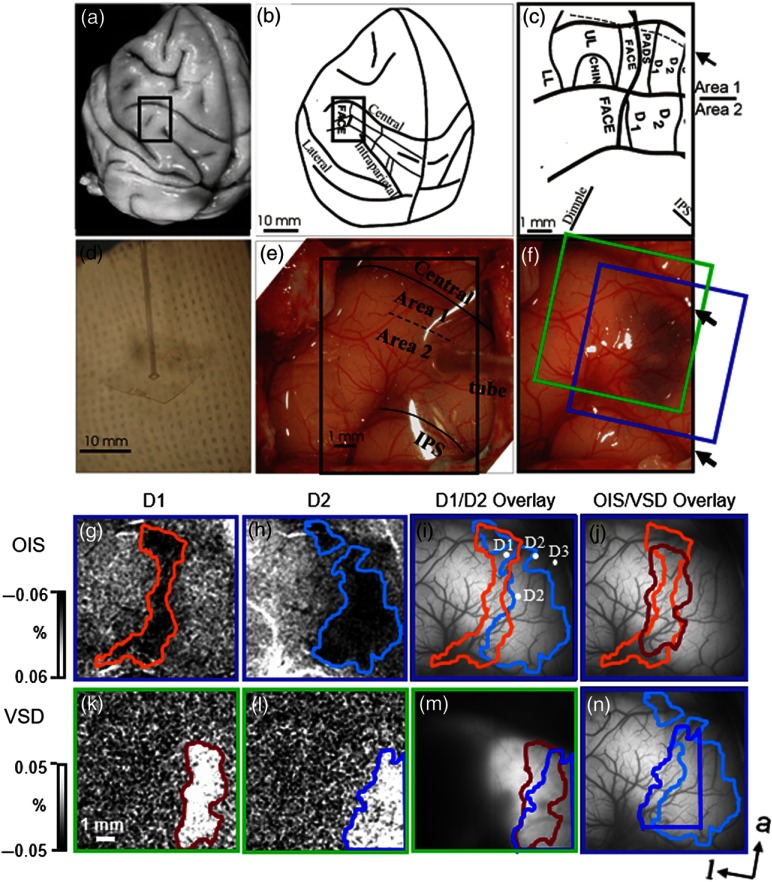
VSD imaging in macaque monkey SI. (a) Boxed region overlies part of the hand and face region of macaque monkey somatosensory cortex. (b), (c) Schematic drawing (from Ref. 23). (d) Dye is infused through plastic tube attached to artificial dura tecoflex. (e) Areas 1 and 2 of macaque somatosensory cortex seen through Tecoflex™ with dye tube attached prior to dye infusion. Central, central sulcus; IPS, intraparietal sulcus. (f) Cortex seen after dye infusion; blue stain is visible. Blue box: region of OIS imaging in (g)—(j). Green box: region of VSD imaging in (k)—(m). (g)–(j) OIS imaging in response to cutaneous stimulation of D1 (g) and D2 (h). (i) Overlay of D1 and D2 activations. Four white dots: electrode recording sites where responses to D1, D2, and D3 were obtained. (j) Overlay of D1 OIS (orange) and D1 VSD (red) activations. (k)–(n) VSD imaging in response to D1 (k) and D2 (l) cutaneous stimulation. (m) Overlay of D1 and D2 VSD activations. (n) Overlay of D2 OIS (light blue) and D2 VSD (dark blue) activations. For OIS, (g) and (h) are the sum of 42 trails and images 1.2 to 2.4 s (see Fig. 2). For VSD, (k) and (l) are the sum of 62 trials and images 130 to 230 ms (see Fig. 2). For (g)–(h) and (k)–(l), spatial resolution 21  μm/pixel. a, anterior; l, lateral. Digit boundaries were based on thresholding and visual inspection; in SI activation, borders do not change dramatically with thresholds ranging from 20% to 80% half maximal amplitude.^18^^,^^20^

We examined the spatiotemporal map of the hand representation in SI in anesthetized macaque monkeys. Previous electrophysiological and optical imaging studies have shown that digits D1 to D5 are represented in a mediolateral fashion in SI of macaque monkeys.^21^^,^^22^ To examine whether VSD methods could reveal such somatotopic maps, we exposed a region of somatosensory cortex overlying areas 1 and 2 in a macaque monkey [Figs. 1(a)–1(c)]. Following brief electrophysiological mapping of a few digit representations [see Fig. 1(i)] to ascertain the location in SI, we conducted OIS in response to vibrotactile stimulation of D1, D2, and D3. We then prepared the cortex for VSD imaging. VSD staining of cortex was achieved by minipump infusion of VSD over 1.5 to 2 h through a tube attached to a piece of artificial dura (Tecoflex™) that covered the cortex [Figs. 1(d) and 1(e)]. This procedure resulted in evident staining of an area roughly 3  mm×6  mm in size [Fig. 1(f)]. Following successful staining of cortex, we conducted VSD imaging during stimulation of individual digits and compared these maps to those obtained with OIS.

We observed spatial similarity between the extents of OIS and VSD activations. OIS revealed the predicted topographic map in areas 1 and 2, consistent with electrophysiological mapping [Fig. 1(i)]. In response to D1 or D2 stimulation [Figs. 1(g) and 1(h)], digit activation appeared as an elongated region overlying areas 1 and 2, roughly 1 mm in width and oriented in the anteroposterior axis. This activation size and orientation is consistent with previous electrophysiological^21^^,^^23^ and optical imaging^22^ studies of digit representation in macaque SI. Stimulation on D1 [Fig. 1(k)] and D2 [Fig. 1(l)] produced similar regions of VSD activation [Fig. 1(m)]. Overlay of the OIS and VSD maps for D1 [Fig. 1(j)] and D2 [Fig. 1(n)] overlapped, although, because some of the D2 activation fell outside the field of view, we are unable to evaluate the degree of similarity. Thus, generally, the VSD activation is topographically appropriate.

**Fig. 2:**
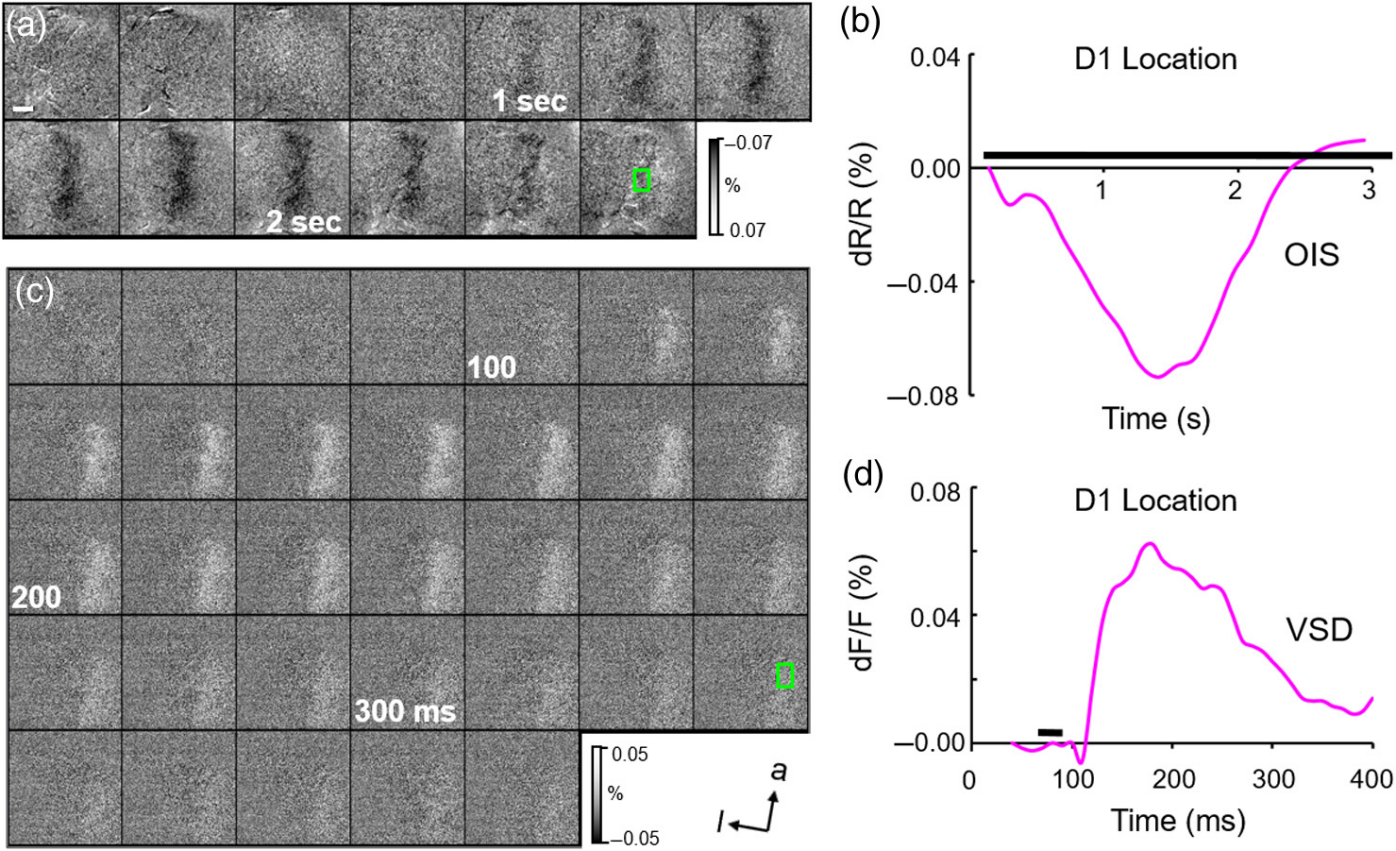
Timecourses of OIS and VSD response. The same case as shown in Fig. 1. (a) OIS images obtained at 5 Hz for 3 s. Images obtained from blue box region in Fig. 1(f). Sum of 42 trials. (b) Plot of negative reflectance change following a 3-s cutaneous stimulus (black bar). ROI: green rectangle in (a). Timecourse and magnitude is typical of OIS response from monkey SI. (c) VSD images obtained at 100 Hz for 400 ms (only 340 ms shown as signal declines to baseline). Images obtained from green box region in Fig. 1(f). Sum of 62 trials. (d) Plot of positive fluorescence change following a 20-ms cutaneous stimulus (black bar above). ROI: green rectangle in (c). Scale bar in (a): 1 mm, applies to all frames in (a) and (c). For (g)—(h), spatial resolution 21  μm/pixel. a, anterior; l, lateral.

We next examined the temporal timecourse of the OIS and VSD signals. OIS images were acquired at 5 Hz for a total of 3 s; VSD images were acquired at 100 Hz for a total of 400 ms. As previously shown, in response to tactile stimulation, the intrinsic signal becomes apparent in the first second, followed by an increasing magnitude of response, which peaks around 2 s and subsequently returns to baseline [Figs. 2(a) and 2(b)]. The VSD signal rises around 100 ms, peaks around 200 ms, and subsequently declines to baseline after 400 ms [Figs. 2(c) and 2(d)] (note that the field of view was moved relative to the initial intrinsic signal imaging) [see Fig. 1(f)].

Thus, both intrinsic signal and VSD signal reveal cortical response to tactile stimulation. The spatial distributions of cortical response assessed with IOS and VSD are similar and suggest that VSD is also capable of revealing topographic cutaneous maps. Under these stimulation conditions, VSD timecourses last for about 200 ms and can be tracked with at least 10-ms resolution. Note that, in comparison with rodents, the timecourse relative to stimulus onset is slower in monkeys (monkey: rise time around 50 ms, peaks around 100 ms, declines after 200 ms; rat: peaks at 10 to 15 ms, declines rapidly by 20 ms, cf. Ref. 20). Moreover, in rats, the VSD signal exhibits rapid lateral spread leading to loss of topographic response within tens of milliseconds, whereas, in monkey, the VSD activation remains spatially confined and topographically appropriate over the signal timecourse. Although, admittedly, the tactile stimulation paradigms in monkeys and rats are different (monkey: vibrotactile stimulation of the skin via 3-mm diameter plastic probe for a single 20 ms, 0.3 mm displacement delivered via piezoceramic actuator; rat: 250- to 300-μm whisker displacement with 20-ms squarewave delivered via piezoceramic actuator), both are robust stimuli. This suggests that these two species exhibit different VSD spatiotemporal timecourses, perhaps indicative of differences in cortical circuitry and organization.

#### 3.1.2 Squirrel monkey

**Fig. 3:**
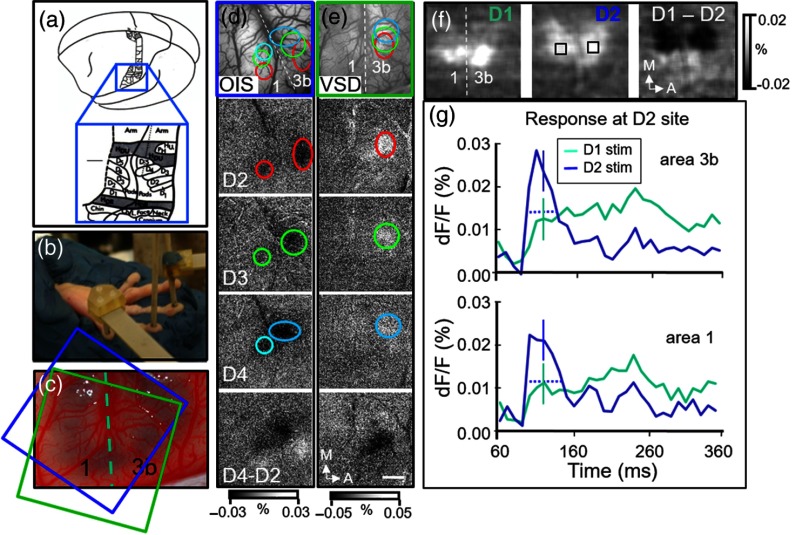
VSD imaging in squirrel monkey SI. (a) Boxed region overlies hand region of squirrel monkey somatosensory cortex (from Ref. 26). (b) Photo of plastic probes positioned on digit tips for delivery of indentation and vibrotactile stimuli. (c) Squirrel monkey cortex (areas 3b and 1) following VSD staining. (d) and (e) OIS and VSD images obtained from case shown in (c). Ovals encircle regions of activation. (d) OIS: Vibrotactile stimulation of D2, D3, and D4 reveal topographic maps in areas 3b and 1. Overlay shown in top panel. Bottom panel: subtraction of D4 and D2. Sum of 44 trials. (e) VSD: Tactile stimulation of D2, D3, and D4 reveal topographic maps in areas 3b and 1. Overlay shown in top panel. Bottom panel: subtraction of D4 and D2. Sum of 50 trials. (f) and (g) VSD imaging from a second case. (f) Imaged response to D1 (left panel) and D2 [middle panel, black boxes are the ROI locations of timecourse response shown in (g)]. Right panel is result of D1–D2 subtraction. Sum of 80 trials. (g) Timecourse of response from (f) from D2 site in areas 3b (top graph) and 1 (bottom graph). Frames obtained at 100 Hz. Blue lines: following stimulation of D2. Green lines: following stimulation of D1. Solid vertical lines: average standard error during the peak response. Dotted horizontal lines: width at half height. Spatial resolution 15.6  μ/pixel; A, anterior; M, medial.

Unlike macaque monkeys, all of the four SI Brodmann areas (3a, 3b, 1, and 2) are visible from the surface in the lissencephalic squirrel monkey^24^ [Fig. 3(a)]. We have previously conducted extensive OIS studies of vibrotactile digit response in areas 3b and 1 in squirrel monkeys^18^^,^^19^^,^^25^ and now examine whether the greater temporal resolution of VSD can offer additional insights.

We first examined the spatiotemporal map of the hand somatotopy in anesthetized squirrel monkeys. We exposed a region of somatosensory cortex overlying areas 3b and 1 and delivered vibrotactile stimuli with plastic probes placed on multiple digit tips, typically three digits within a single run [Fig. 3(b)]. Following intrinsic signal imaging of digit maps, we then stained the cortex with VSD [Fig. 3(c)]. In this case, we placed a volume of VSD on the exposed cortex and, with occasional replacement, allowed staining to occur over 1.5 to 2 h. This procedure was effective in roughly 70% of attempts.

We observed spatial similarity between the extents of OIS and VSD activations. OIS revealed the predicted topographic map in area 3b, revealing a lateral to medial progression of D2, D3, and D4 [Fig. 3(d), outlined in red, green, and blue, respectively; overlay shown in top panel; bottom panel illustrates subtraction of D4, dark pixels, and D2, light pixels]. Also visible is a weaker but parallel topographic map in area 1 [Fig. 3(d)]. VSD imaging of the same region with stimulation of D2, D3, and D4 produced similar regions of activation in area 3b, although, in this case, relatively weaker signals in area 1 [Fig. 3(e), outlined in red, green, and blue, respectively; overlay shown in top panel; bottom panel illustrates subtraction of D4, light pixels, and D2, dark pixels]. In another case, comparable activations in areas 3b and 1 were obtained with VSD imaging [Fig. 3(f)]. In both areas 3b [Fig. 3(g), upper] and 1 [Fig. 3(g), lower], the timecourse of these activations at the cortical D2 site was, as expected, robust to stimulation on digit D2 (blue) but not digit D1 (green), indicating selective topographic activation. Also consistent with OIS, the magnitude of activation in area 1 (lower, blue) was weaker than that in area 3b (upper, blue). Note that the VSD response duration was 40 ms (area 3b) and 45 ms (area 1) at half height [dotted lines in Fig. 3(g)], comparable to the 20-ms indentation stimulus duration.

In sum, consistent with OIS mapping, VSD imaging reveals topographic cortical response to vibrotactile stimulation. These topographic maps are revealed in both Old World macaque monkeys and New World squirrel monkeys. The spatial distributions of cortical response assessed with IOS and VSD are generally similar. Under these stimulation conditions, VSD signals are comparable in duration to stimulus presentation periods and can be tracked with at least 10-ms resolution.

### 3.2 Can Voltage-Sensitive Dye Reveal Inhibitory Processes in Monkey SI?

We have previously examined the cortical representation of tactile funneling, a percept in which dual point skin stimulation leads to a perceived merged stimulus location in between the actual sites of stimulation.^18^^,^^19^ We demonstrated that, in response to two point skin stimulation, activation in area 3b and in area 1 of squirrel monkey SI parallels the percept. That is, a single site of cortical response in between the single activation sites is observed, one which is stronger in magnitude than that at either site alone, consistent with the funneling percept.^27^ This phenomenon and associated neurophysiological studies^28^^,^^29^ predict that the inputs that arrive at the cortical locations corresponding to the actual stimulated skin sites are relatively suppressed, perhaps via subthreshhold mechanisms. We now examine whether such changes in subthreshhold activity can be inferred with VSD methods.

**Fig. 4:**
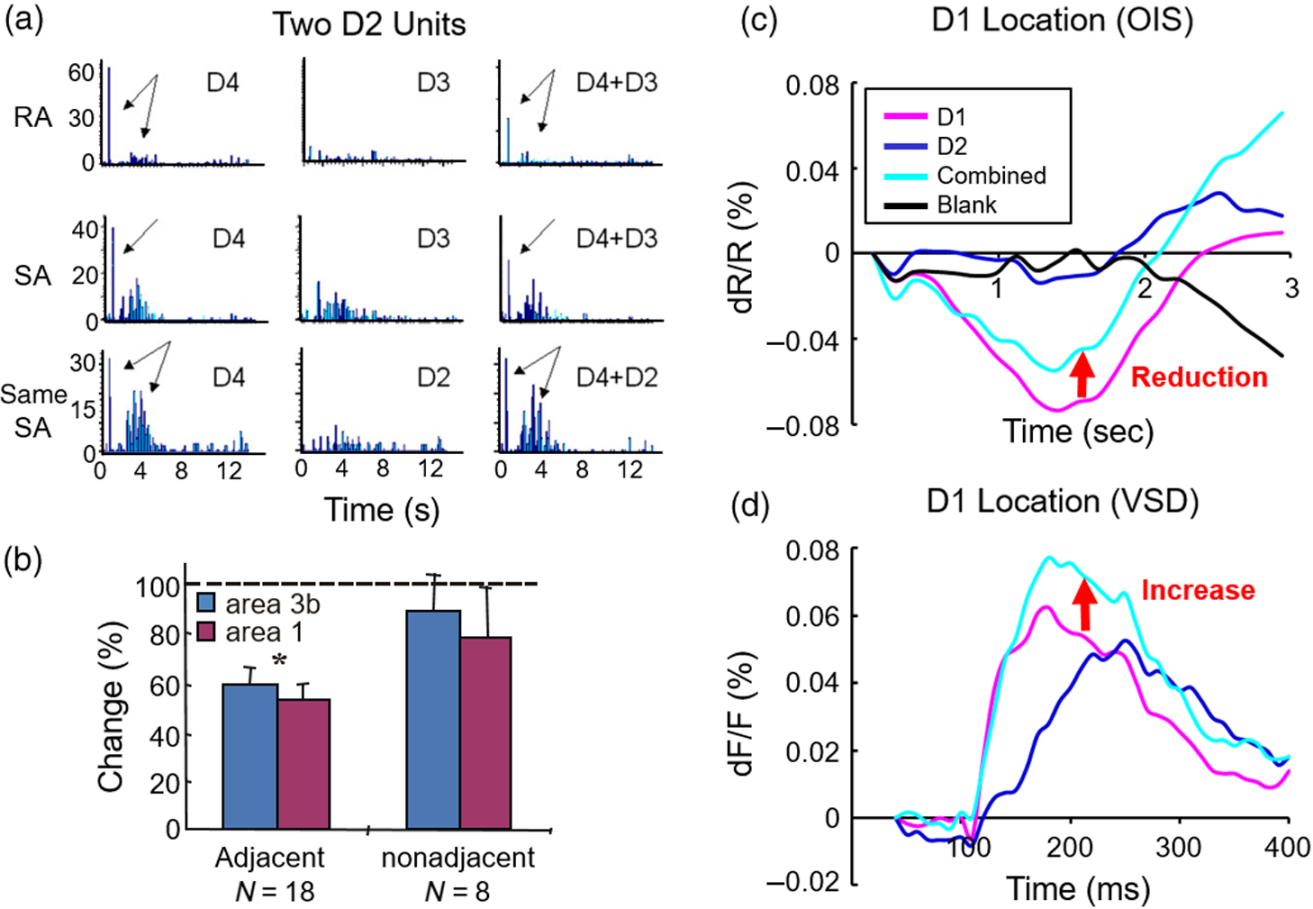
Tactile funneling response in monkey SI. (a) Two single units recorded in squirrel monkey area 3b. RA unit: top row. SA unit: bottom two rows. See text. (b) Summary of units recorded in areas 3b and 1, illustrating suppressive effects of paired adjacent digits but not nonadjacent digits (from Ref. 19). (c) Timecourse of OIS from macaque area 1 of D1 (pink), D2 (dark blue), and D1+D2 stimulation (cyan), and no stimulation (blank, black). ROI centered in D1 is shown in Fig. 2(b). The same case as Figs. 1 and 2. Sum of 42 trials. At the D1 location, paired stimulation produces a relative suppression compared with single digit D1 stimulation (red arrow). (d) VSD recording of D1 (pink), D2 (dark blue), and D1+D2 stimulation (cyan). ROI within the D1 representation is shown in Fig. 2(d). Sum of 62 trials. At the D1 location, in comparison with single digit D1 stimulation, paired stimulation produces a relative enhancement of VSD signal (red arrow). Note that being unable to synchronize to heart rate and respiration for this experiment, the standard error measurements for the OIS (c) and VSD (d) time courses were relatively large, 0.04% and 0.017%, respectively.

We briefly summarize electrophysiological results from a previous study on cortical tactile funneling,^19^ which provides context to our current OIS and VSD results. Figure 4(a) shows electrophysiological recordings from two units in squirrel monkey area 3b; one is a rapidly adapting (RA) unit (top row) and the other is a slowly adapting (SA) unit (same unit bottom two rows). The RA unit’s receptive field is located on D4, as evidenced by the large early transient response to probe indentation on D4 (top row, left) and lack of response to D3 indentation (top row, middle). Simultaneous stimulation of adjacent digits (D4 and D3) leads to a suppression of response (top row, right). The SA unit exhibits initial transient response to D4 and a later more sustained response to stimulation of either D4 or D3 (middle row, left, and middle). Simultaneous stimulation of D4 and D3 produces a marked decrease in the transient component of this SA unit, although no change in the sustained component (middle row, right). Consistent with previous studies on tactile funneling,^18^^,^^19^^,^^28^^,^^29^ this two digit suppression is observed on adjacent digits (D4 and D3), but not on nonadjacent digits (D4 and D2, bottom row). As summarized in Fig. 4(b) (from Ref. 19), these data support the presence of local intracortical suppression mediated via local lateral cortical circuits and indicate that their extent is limited to adjacent digits.

We then examined the cortical funneling response with intrinsic OIS in the macaque monkey (same case as Fig. 1). We observed [Fig. 4(c)] that, at the cortical representation of D1 in area 1, stimulation of D1 (pink) produces a robust negative reflectance change, whereas stimulation of D2 (dark blue) produces little response, as would be expected from a topographic response. When D2 and D1 are stimulated together (cyan), a reduction in reflectance change is observed (red arrow, reduction of OIS magnitude). These results are consistent with our electrophysiological results and with previous optical imaging findings in squirrel monkeys.^18^^,^^19^

At the same location in area 1, following VSD staining, we find that VSD signals have a somewhat different response profile [Fig. 4(d)]. In response to D1 indentation alone (pink), the VSD signal reaches a peak within 200 ms and remains elevated over the next 200 ms. Unlike OIS, stimulation of D2 alone (dark blue) leads a prominent VSD response of similar magnitude, which is delayed by roughly 80 to 100 ms. Thus, VSD detects the presence of a nontopographic response, one that is substantially delayed and is potentially due to transmission via lateral cortical circuits. Moreover, when D2 and D1 are stimulated together (cyan), the response is increased rather than decreased (red arrow, increased VSD magnitude). This finding, in combination with the concomitant suppression observed with electrophysiology and optical imaging, suggests that VSD is detecting suppressive subthreshhold contributions, as proposed by previous funneling studies.^18^^,^^28^^,^^29^

To summarize, although OIS reveals signal suppression with dual digit stimulation [red arrow in Fig. 4(c)], VSD shows an enhanced signal [red arrow in Fig. 4(d)]. This indicates that: (1) OIS correlates more with the spiking activity than VSD (consistent with previous studies)^3^^,^^4^ and (2) VSD reveals previously hypothesized inhibitory effects induced by dual digit stimulation.^18^^,^^29^ Although this may not be surprising, it has not been previously demonstrated with VSD imaging methods in monkeys. The comparison between OIS and VSD for examining such digit integration has also not been previously shown. VSD imaging extends our understanding of cortical encoding of tactile funneling and further suggests the presence of suppressive intracortical mechanisms. Our findings also suggest that subthreshhold contribution is a more dominant part of the VSD signal than the OIS signal.

### 3.3 Can Voltage-Sensitive Dye Be Used to Reveal Motion Selectivity in Monkey SI?

An important finding regarding monkey SI is that area 1 may be specialized for processing cutaneous motion information. Neurons in area 1 are reported to have stronger selectivity for cutaneous motion direction than area 3b^30^ (cf. Refs. 31–33). Pei et al.^30^ found that area 1 neurons are tuned to the direction of both continuous tactile sweeps and apparent motion generated by rapid sequential stimulation of skin locations, stimuli that produce salient percepts of cutaneous motion. This report of motion invariance in area 1 provided strong support for area 1’s role in motion direction processing. It is also known that these effects are not peripheral and are cortically generated.^34^ Given the spatiotemporal nature of motion stimuli, we now examine whether areal distinctions may be revealed using VSD imaging in squirrel monkey SI.

**Fig. 5:**
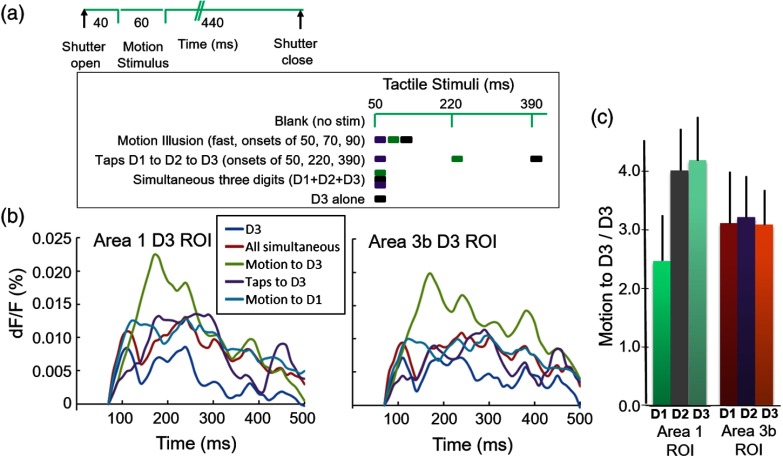
VSD study of apparent motion response in squirrel monkey SI. (a) Top: timing of image acquisition and tactile stimulus presentation. Inset: five different tactile stimulus conditions were presented. (b) Timecourse of response from a D3 site in area 1 (left graph) and area 3b (right graph). Profiles: green (motion to D3): D1-to-D2-to-D3 stimulation presented at 50, 70, and 90 ms, each a 20-ms tap. Light blue (motion to D1): D3-to-D2-to-D1 stimulation presented at 50, 70, and 90 ms. Purple (taps to D3): 20-ms taps presented to D1-to-D2-to-D3 at 50, 220, and 390 ms. Red (all simultaneous): D1, D2, and D3 stimulated for 20 ms simultaneously. Green (D3 alone): single digit D3 stimulated for 20 ms. Frames obtained at 100 Hz. Sum of 42 trials. (c) Comparison of motion response relative to static stimulus response. Analysis done for each of the three digit representation locations: area 1: D1 (bright green), D2 (gray), and D3 (muted green); area 3b: D1 (red), D2 (purple), and D3 (orange). Response magnitude difference (the difference between the “motion to D3” value and the “D3” value within the 100- to 400-ms time windows) normalized by stimulus to D3 alone. Solid vertical lines: SEM.

Following cortical staining of squirrel monkey SI, we presented cutaneous motion stimuli for 60 ms (images acquired at 100 Hz for 500 ms). Imaging and tactile stimulation timing is shown in Fig. 5(a). We used apparent motion stimuli in which a rapid sequence of 20-ms taps were presented sequentially to D1, D2, and D3 at 50, 70, and 90 ms, respectively. As shown in Fig. 5(b) timecourses, two directions of apparent motion were presented: toward D3 (D1-to-D2-to-D3) (green) and away from D3 (D3-to-D2-to-D1) (light blue). These motion stimuli were compared to: (1) slower sequential 20-ms taps (presented at 50, 220, and 390 ms) that do not induce a motion percept, toward D3 (D1-to-D2-to-D3) (purple), (2) simultaneous stimulation of the three digits (duration 20 ms) (red), (3) indentation of D3 alone (duration 20 ms) (dark blue), and (4) no stimulation condition (subtracted from each of previous conditions). Each of these conditions was presented in randomly interleaved fashion for 45 trials.

As shown in Fig. 5(b) (left), at the location of D3 in area 1, all the tactile stimulation conditions produced a fluorescence response of roughly 0.025% or less, rose rapidly between 50 and 100 ms after stimulus onset, and returned to baseline within about 300 ms. Our first observation was that, at the cortical D3 site, of the stimulus conditions presented, the strongest response was to the apparent motion stimulus toward D3 (green, D1-to-D2-to-D3, peak amplitude in first 200 ms). This motion response was 20% larger in area 1 than in area 3b [compare with Fig. 5(b), right]. Importantly, the response was almost twice as large to the apparent motion stimulus “toward D3” direction as the “away from D3” direction (D3-D2-D1, light blue), demonstrating a directionally selective, population-based “toward digit” response in both areas 3b and 1.

This “toward digit” response in both areas 1 and 3b was much larger in amplitude than all other conditions. The next largest responses were the motion response away from the digit (light blue), the slower sequential tapping condition toward D3 (purple), and the simultaneous all digit condition (red). The single D3 indentation was weakest among these conditions (dark blue). The response profiles for the motion stimuli are qualitatively similar in areas 3b and 1: both areas 3b and 1 exhibit greater response for motion than other static and motion conditions (slow sequential taps toward the digit, motion toward other digits, e.g., D1).

To further examine whether this directionally selective response is in any way specific to the cortical representation of D3, we compared motion versus nonmotion stimulus response at D1, D2, and D3 cortical sites. As shown in Fig. 5(c), normalizing the “apparent motion to D3” response by the “static D3 indentation” produces a much greater motion preference in area 1 than area 3b at the D3 and D2 sites than the D1 site. In contrast, area 3b only showed a nonspecific somatotopic response to the apparent motion stimulus, consistent with multidigit interactions previously described in area 3b.^28^^,^^35^ That is, area 1 shows greater digit-specific response: it distinguishes among D1, D2, and D3 in its response magnitude “toward the digit of interest” (in this case, motion toward D3), whereas area 3b exhibits similar responses at all digit locations for the “motion toward D3” response. This may suggest a digit selectivity for this motion stimulus in area 1 and is consistent with the greater digit integration at adjacent than nonadjacent sites, demonstrated in the tactile funneling illusion. Although further investigation is required to establish the strength of this finding, it may suggest that in area 1 at the population level, there is directional selectivity and this selectivity is relative to topographic location.

These findings suggest that, in area 1, there is a population-based preference for motion stimuli over static stimuli, a directional preference of motion stimulation toward the represented digit location, and, furthermore, a velocity dependence of spatial integration (faster better than slower, cf. Ref. 36), consistent with a cortical response underlying the cutaneous apparent motion percept.

## 4 Discussion

### 4.1 Summary

We report that VSD imaging is a very useful tool for studying activation in monkey SI in response to cutaneous stimuli. In both macaque and squirrel monkeys, observed somatotopy and extent of single digit activation were quite similar to that mapped with OIS. In response to single or two digit indentation, when tracked with 10-ms resolution, evolution of the fluorescence signal could be detected as early as 25 to 40 ms poststimulus onset, climbed rapidly within 10 to 20 ms, and remained elevated for 100 to 300 ms; dF/F signal sizes fell in the 0.01% to 0.1% range [Figs. 2(c)–2(d), 3(e)–3(g), 4(d)]. In response to sequential apparent motion stimulation consisting of three 20-ms taps on three adjacent digits, the VSD signal followed comparable timecourses [Fig. 5(b)]. Thus, VSD is capable of tracking somatosensory cortical activity with high spatial and temporal resolution.

### 4.2 Comparison to Rodent Somatosensory Cortex

VSD studies of rodent somatosensory cortex have revealed topographic maps of facial vibrissae (e.g., Refs. 20, 37–40). In response to single whisker deflection, these signals are characterized by focal barrel-specific activation within the first 10 to 20 ms followed by rapid, extensive, and anisotropic spread across barrel cortex.^20^ Consistent with and extending electrophysiological recordings, VSD studies of distant whisker deflections and multiwhisker activation revealed the presence of subthreshold modulatory responses and complex intracortical interactions (e.g., Refs. 37, 41–43). We note that, unlike rodent barrel cortex, the VSD signal in monkey SI remains topographically confined. This could indicate differences in lateral network signal propagation and/or differences in relative balance of spiking versus subthreshhold response.

### 4.3 Study of Multidigit Integration

Our tactile funneling results suggest that there are subthreshhold modulatory influences induced by adjacent two digit tactile stimulation. Whereas both spiking and OIS mapping reveal a reduction in response by two digit versus one digit stimulation, VSD signals increase. This is consistent with VSD’s detection of subthreshhold influences induced by inhibitory lateral influences and adds to existing electrophysiological and OIS literature on the cortical basis of tactile funneling.

Using VSD, we show for the first time evidence for cutaneous motion selective response at the population level in monkey areas 3b and 1. We find that both areas 3b and 1 exhibit greater response to apparent motion than to static nonmotion stimuli [Fig. 5(b)]. Our data thus far suggest that area 1 may be distinguished from area 3b in that, at the recorded cortical location, this motion response is directionally selective toward the represented digit location [Fig. 5(c)]. What this “toward digit” preference signifies with respect to hand use remains to be further studied. Further studies are needed to examine the thesis that area 1 is specialized for processing cutaneous motion direction information.^30^

### 4.4 Future Studies

Primate hand use is characterized by the coordinated use of multiple digits in power grasp and fine precision grip. These different types of manual behavior are accompanied by distinct patterns of cutaneous inputs from the hand. We are encouraged by the results obtained using VSD imaging in monkey somatosensory cortex. These results indicate that VSD imaging constitutes a new tool for studying somatosensory processing and multidigit integration. We believe the possibility of using VSD to examine cortical mechanisms underlying multidigit integration during grasp behavior in the awake monkey is promising.
